# Profiling of Viral Proteins Expressed from the Genomic RNA of Japanese Encephalitis Virus Using a Panel of 15 Region-Specific Polyclonal Rabbit Antisera: Implications for Viral Gene Expression

**DOI:** 10.1371/journal.pone.0124318

**Published:** 2015-04-27

**Authors:** Jin-Kyoung Kim, Jeong-Min Kim, Byung-Hak Song, Sang-Im Yun, Gil-Nam Yun, Sung-June Byun, Young-Min Lee

**Affiliations:** 1 Department of Animal, Dairy, and Veterinary Sciences; Utah Science Technology and Research, College of Agriculture and Applied Sciences, Utah State University, Logan, Utah, United States of America; 2 Department of Microbiology, College of Medicine, Chungbuk National University, Cheongju, South Korea; 3 Animal Biotechnology Division, Korea National Institute of Animal Science, Suwon, South Korea; Wuhan University, CHINA

## Abstract

Japanese encephalitis virus (JEV), a mosquito-borne flavivirus, is closely related to West Nile (WN), yellow fever (YF), and dengue (DEN) viruses. Its plus-strand genomic RNA carries a single open reading frame encoding a polyprotein that is cleaved into three structural (C, prM/M, and E) and at least seven nonstructural (NS1/NS1', NS2A, NS2B, NS3, NS4A, NS4B, and NS5) proteins, based on previous work with WNV, YFV, and DENV. Here, we aimed to profile experimentally all the viral proteins found in JEV-infected cells. We generated a collection of 15 JEV-specific polyclonal antisera covering all parts of the viral protein-coding regions, by immunizing rabbits with 14 bacterially expressed glutathione-*S*-transferase fusion proteins (for all nine viral proteins except NS2B) or with a chemically synthesized oligopeptide (for NS2B). In total lysates of JEV-infected BHK-21 cells, immunoblotting with these antisera revealed: (*i*) three mature structural proteins (~12-kDa C, ~8-kDa M, and ~53-kDa E), a precursor of M (~24-kDa prM) and three other M-related proteins (~10-14 kDa); (*ii*) the predicted ~45-kDa NS1 and its frameshift product, ~58-kDa NS1', with no evidence of the predicted ~25-kDa NS2A; (*iii*) the predicted but hardly detectable ~14-kDa NS2B and an unexpected but predominant ~12-kDa NS2B-related protein; (*iv*) the predicted ~69-kDa NS3 plus two major cleavage products (~34-kDa NS3^N-term^ and ~35-kDa NS3^C-term^), together with at least nine minor proteins of ~16-52 kDa; (v) the predicted ~14-kDa NS4A; (*vi*) two NS4B-related proteins (~27-kDa NS4B and ~25-kDa NS4B'); and (*vii*) the predicted ~103-kDa NS5 plus at least three other NS5-related proteins (~15 kDa, ~27 kDa, and ~90 kDa). Combining these data with confocal microscopic imaging of the proteins’ intracellular localization, our study is the first to provide a solid foundation for the study of JEV gene expression, which is crucial for elucidating the regulatory mechanisms of JEV genome replication and pathobiology.

## Introduction

Japanese encephalitis virus (JEV) is an emerging pathogen that belongs to the genus *Flavivirus* in the family *Flaviviridae* [1,2]. Within the genus, JEV is a member of the JE serogroup that also includes three other medically important pathogens, i.e., West Nile virus (WNV), St. Louis encephalitis virus, and Murray Valley encephalitis virus [3]. JEV also bears a close genetic relationship to other clinically significant flaviviruses: e.g., yellow fever virus (YFV), dengue virus (DENV), and tick-borne encephalitis virus (TBEV) [4]. JEV is the etiological agent of JE [5], a serious neurological disease that occurs commonly across much of Asia [6–8] and appears sporadically in northern Australia and parts of the Western Pacific islands [9–11]. Although JE incidence is low in some JE-endemic areas (e.g., Japan, South Korea, and Taiwan), it is significantly high and appears to be rising in many others (e.g., Bangladesh, Cambodia, Indonesia, and Pakistan) [12]. Notably, large JE outbreaks have occurred during the past decade in India and Nepal [13–16], underscoring the fact that JE remains a major public health problem in Asia. Moreover, JE emerged during the 1990s in Papua New Guinea and in the Torres Strait islands, from which it spread to the Cape York Peninsula in Australia [10,11,17], raising concerns that JE may continue to appear in new areas around the world [12,18–21].

JEV is an arbovirus that circulates in a natural enzootic cycle between vertebrate hosts and mosquito vectors [22,23]. Although many animals are susceptible to JEV infection, pigs and birds act as the major hosts important for viral amplification, maintenance, and dissemination [24]. A pool of different mosquito species may serve as vectors for JEV transmission, but culicine mosquitoes, especially *Culex tritaeniorhynchus*, are the primary mosquito species associated most strongly with JEV infection in humans [25,26]. Humans are accidental dead-end hosts, because they do not develop a level or duration of viremia sufficient to transmit JEV to mosquitoes [27]. The vast majority of JEV-infected individuals are asymptomatic; only 1 in 25–1,000 infections develops into clinical symptoms [27–30], with an average incubation period of 5 to 15 days [31]. JEV infection may cause a broad spectrum of diseases [27], ranging from a mild febrile illness to aseptic meningitis and severe encephalitis [32–38]. The global incidence of JE is not precisely known, but about 50,000–175,000 cases are estimated to occur annually in JE-endemic areas, depending on geographic location, age group, and vaccination status [8,39,40]. Approximately 20–30% of JE patients die, and up to 50% of JE survivors develop long-term neurologic and psychiatric deficits [8,39,41]. Thus, the clinical burden of JE is substantial, and despite the fact that four JEV vaccines are licensed in various parts of the world [24,42–44], there is still no specific treatment available for JEV infection [45–47].

JEV is a ~50-nm-sized enveloped virion [25] containing an ~11-kb-long, plus-strand RNA genome that is 5'-capped and 3'-nonpolyadenylated [48,49]. The genomic RNA encodes a single long open reading frame (ORF) between the 5' and 3' short non-coding regions (NCRs) [50–52] that are important for RNA replication [53,54]. Like other flaviviruses, JEV enters its host cells via receptor-mediated endocytosis [55–57]. In an acidic endosomal compartment, the viral membrane fuses with the host membrane, and the viral genome is then released into the cytoplasm [58–60]. The ORF encoded in the genome is translated into a polyprotein, which is cleaved by host and viral proteases to produce at least 10 individual proteins [25,61]: NH_2_-C-prM-E-NS1-NS2A-NS2B-NS3-NS4A-NS4B-NS5-COOH [62]. The three N-terminal structural proteins are components of viral particles [63,64], and the seven C-terminal nonstructural proteins participate in multiple events during viral replication: RNA genome replication [65–70], virus particle assembly [71–75], and innate immune evasion [76–78]. For members of the JE serogroup, an elongated form of NS1 (NS1') is expressed by -1 ribosomal frameshifting at codons 8–9 of NS2A, adding 52 extra amino acids to the C-terminus of NS1 [79,80]. RNA replication is catalyzed by the two multifunctional nonstructural proteins: (***i***) NS3 possesses serine protease (with its cofactor NS2B), helicase, nucleoside triphosphatase (NTPase), and RNA triphosphatase (RTPase) activity [81–85]; and (***ii***) NS5 has methyltransferase (MTase), guanylyltransferase (GTase), and RNA-dependent RNA polymerase (RdRP) activity [86–90]. Viral assembly occurs by budding of the viral genomic RNA and C proteins into the endoplasmic reticulum, where the budding particles acquire a lipid bilayer in which the prM and E proteins are embedded [91–93]. These immature virions are transported to the cell surface through the secretory pathway [94]; in the *trans*-Golgi network, prM on the viral membrane is cleaved by furin to yield the mature M protein [95,96], converting the immature non-infectious particle to a mature infectious virion [97].

Despite the global impact of JE, our current knowledge of JEV biology remains limited because of the lack, or limited availability, of JEV-specific antibodies, which poses a technical challenge for the detection and functional studies of JEV gene products. In the present study, we have generated a full set of 15 JEV antigen-specific rabbit polyclonal antisera that cover all parts of the viral protein-coding regions. Using these antisera, we have performed a large set of immunoblot analyses, enabling us to identify all the viral gene products accumulated in JEV-infected cells. Also, we have done a full series of confocal microscopy analyses to visualize the intracellular localization of the viral proteins. Our data provide the first comprehensive expression profile of JEV gene products, which is important for advancing our understanding of JEV genome replication and pathobiology.

## Materials and Methods

### Cells and viruses

BHK-21 cells were cultivated in alpha minimal essential medium supplemented with 10% fetal bovine serum, 2 mM L-glutamine, vitamins, and antibiotics [98]. All cell culture reagents were purchased from Gibco (Gibco-Life Technologies, Carlsbad, CA). The JEV strain used in this study was CNU/LP2 [98], a plaque-purified clone of the wild-type pathogenic K87P39 isolate obtained from a pool of *Culex tritaeniorhynchus* mosquitoes in South Korea in 1987 [48,99]. A virus stock was prepared by infecting BHK-21 cells at a multiplicity of infection (MOI) of 1 and harvesting culture supernatants at 3–4 days post-infection when destruction of the cell monolayers was clearly observed.

### Plasmid construction

A total of 16 bacterial expression plasmids were constructed, each of which was used to express a small non-hydrophobic region of the JEV polyprotein as a glutathione *S*-transferase (GST) fusion protein. In all 16 plasmids, a defined region of the JEV ORF was first amplified by PCR using the full-length infectious cDNA molecular clone of JEV CNU/LP2 (pBAC^SP6^/JVFLx/XbaI [98]) as a template and the appropriate pair of primers listed in Table 1 (note that primer names indicate the target viral protein-coding region, followed by the orientation of each primer, forward (f) or reverse (r)). Next, the resulting PCR amplicons were individually ligated in-frame to the 3'-end of GST coding sequence in the pGex-4T-1 vector (GE Healthcare, Piscataway, NJ) using either the *Eco*RI and *Xho*I sites (for the first 14 PCR amplicons except NS5^N-term^ and NS5^C-term^) or the *Bam*HI and *Xho*I sites (for NS5^N-term^ and NS5^C-term^). The nucleotide sequences of the cloned cDNAs were verified by DNA sequencing.

**Table 1:** Oligonucleotides used for PCR amplification and cDNA cloning.

Oligonucleotide	Sequence^*a*^ (5' to 3')	Position^*b*^	Polarity
Cf	ccggaattcATGACTAAAAAACCAGGAGG	96–115	Sense
Cr	ccgctcgagctattaGTTTTGCTTTCTGCCCCGCT	385–404	Antisense
PRf	ccggaattcATGAAGTTGTCAAATTTCCA	477–496	Sense
PRr	ccgctcgagctattaCCTCCTGCTTCGCTTGGAAT	733–752	Antisense
Mf	ccggaattcTCCGTGTCGGTCCAAACACA	753–772	Sense
Mr	ccgctcgagctattaGAAAGCATAGCCAGGATTCC	865–884	Antisense
ENf	ccggaattcTTTAATTGTCTGGGAATGGG	978–997	Sense
ENr	cgactcgagctattaACATGTGTCAATGCTTCCCT	1306–1325	Antisense
ECf	ccggaattcATGACCGTGGGGTCAAAGTC	1587–1606	Sense
ECr	cgactcgagctattaTGTACACATGCCATAGGTTG	1873–1892	Antisense
NS1Nf	ccggaattcGACACTGGATGTGCCATTGA	2478–2497	Sense
NS1Nr	ccgctcgagctattaACGGGTTGATGTGATACCAA	2956–2975	Antisense
NS1Cf	ccggaattcGTGTGGCTGAAGATTAGAGA	2976–2995	Sense
NS1Cr	ccgctcgagctattaAGCATCAACCTGTGATCTGA	3514–3533	Antisense
NS1'FSf	ccggaattcTCAGCTGGGCCTTCTGGTGA	3560–3579	Sense
NS1'FSr	ccgctcgagttactaGTGTAAGTGATGCCCCCAAG	3669–3688	Antisense
NS2Af	ccggaattcTTCAATGGTGAAATGGTTGA	3534–3553	Sense
NS2Ar	ccgctcgagttatcaTAGCACCACATACCTCGCCA	3694–3713	Antisense
NS2Bf	ccggaattcGTGTCAGGAAAAGCAACAGA	4347–4366	Sense
NS2Br	ccgctcgagctattaGCAAGACATGCGCAAGACCC	4507–4526	Antisense
NS3Nf	ccggaattcGGGGGCGTGTTTTGGGACAC	4608–4627	Sense
NS3Nr	ccgctcgagctattaTCCTCGTGCGGCTATGCTGG	5485–5504	Antisense
NS3Cf	ccggaattcTACATCGCTACCAAGGTGGA	5505–5524	Sense
NS3Cr	ccgctcgagctattaTCTCTTTCCTGCTGCAAAGT	6445–6464	Antisense
NS4Af	ccggaattcTCGGCCGTTAGCTTCATAGA	6465–6484	Sense
NS4Ar	ccgctcgagctattaTGTGATGGTTTCCAGTGCAT	6610–6629	Antisense
NS4Bf	ccggaattcGCCACTGATGTGCCTGAACT	7377–7396	Sense
NS4Br	ccgctcgagttatcaACTGGCTCCATTATCCCACA	7537–7556	Antisense
NS5Nf	cgtggatccGGAAGGCCTGGGGGCAGGAC	7677–7696	Sense
NS5Nr	ccgctcgagctattaTCCTCGCAGATGGTTTTCCC	8998–9017	Antisense
NS5Cf	cgtggatccGAGTGTCACACATGTATCTA	9018–9037	Sense
NS5Cr	ccgctcgagttactaGATGACCCTGTCTTCCTGGA	10372–10391	Antisense

^*a*^ JEV-specific sequences are shown in uppercase letters, and JEV-unrelated sequences are indicated in lowercase letters. Restriction enzyme recognition sites used for DNA cloning are underlined.

^*b*^ Nucleotide position refers to the complete genome sequence of JEV CNU/LP2 (GenBank accession number AY585243).

### Expression of GST-tagged fusion proteins

A fresh overnight culture derived from a single colony of *E*. *coli* BL21, carrying each of our pGex-based GST fusion protein expression vectors, was diluted 50-fold in 500 ml of LB medium containing 100 μg/ml ampicillin and then incubated with shaking at 35°C until the OD_600_ reached 0.6 to 1.0. To induce the expression of GST fusion proteins, the bacterial culture was further incubated in the presence of 0.1 mM isopropyl β-D-1-thiogalactopyranoside (IPTG) at 35°C for 1–2 h. The optimal induction parameters were determined experimentally for a particular GST fusion protein. Following IPTG induction, cells were harvested by centrifugation at 3,107 × *g* at 4°C for 15 min, followed by washing once with 50 ml of ice-cold phosphate-buffered saline (PBS pH 7.4; 137 mM NaCl, 2.7 mM KCl, 10 mM Na_2_HPO_4_, and 1.8 mM KH_2_PO_4_). The cell pellet was resuspended in 25 ml of ice-cold PBS supplemented with 0.01% Triton X-100 and 1× complete protease inhibitor cocktail (Roche Diagnostics, Indianapolis, IN), and then kept on ice for 30 min with gentle shaking every 5 min. Cell lysis was achieved by sonication on ice, using a S-450D ultrasonic cell disruptor (Branson Ultrasonics, Danbury, CT), until the sample was no longer viscous. The total sonication time was ~15 min, alternating 30-sec pulses with 30-sec pauses. After sonication, the cell lysate was cleared by centrifugation twice at 17,418 × *g* at 4°C for 20 min, and the supernatant (soluble fraction) was immediately used for the purification of GST fusion proteins.

### Purification of GST-tagged fusion proteins

Soluble GST fusion proteins were purified directly from the pre-cleared bacterial lysate by affinity chromatography using the glutathione-Sepharose 4 Fast Flow matrix (GE Healthcare), according to the manufacturer’s instructions. In brief, 2 ml of the 50% glutathione-Sepharose 4 Fast Flow slurry was transferred to a disposable 5-ml polypropylene gravity-flow column (Pierce, Rockford, IL) and washed with 10 bed volumes of ice-cold PBS-T buffer (PBS containing 0.01% Triton X-100 and 1× complete protease inhibitor cocktail, Roche Diagnostics). The equilibrated column was loaded with ~25 ml of the pre-cleared bacterial lysate containing a GST-tagged fusion protein of interest. The column was washed with >20 bed volumes of the ice-cold PBS-T until the OD_280_ of the flow-through was below 0.01. The GST-tagged proteins were then eluted from the affinity matrix with freshly made elution buffer (30 mM glutathione, 50 mM Tris pH 8.8, and 200 mM NaCl). The eluates were collected in 15 tubes (10 drops/tube) and monitored by OD_280_ for protein concentration. All purification procedures were carried out at 4°C. Aliquots (5–10 μl) of each fraction were monitored by sodium dodecyl sulfate-polyacrylamide gel electrophoresis (SDS-PAGE) to verify the integrity and concentration of the fusion protein of interest.

### Production of rabbit polyclonal antisera

We raised a total of 15 polyclonal antisera in rabbits: 14 immunized with each of the bacterially expressed GST fusion proteins and one immunized with a 12-amino-acid synthetic peptide coupled to keyhole limpet hemocyanin (KLH). All of the polyclonal antisera were generated in female New Zealand White rabbits (1.7–1.9 kg) over an 8-week period according to conventional procedures. Two rabbits were immunized with each immunogen. Each rabbit was simultaneously injected twice, both subcutaneously into the back of the neck and intramuscularly in the thigh; each injection consisted of a well-mixed suspension containing 250 μl of complete Freund’s adjuvant (Sigma, St. Louis, MO) and 250 μl of either GST-tagged fusion protein (~100 μg) or KLH-conjugated synthetic peptide (~200 μg). Two weeks after the initial immunization, five consecutive booster immunizations were given weekly with the same fusion protein or synthetic peptide antigen formulated with incomplete Freund’s adjuvant (Sigma). Rabbit sera were collected 5 days after the last boost.

### Ethics statements

All animal experiments were conducted strictly in accordance with the regulations in the Guide for the Care and Use of Laboratory Animals issued by the Ministry of Health and Welfare of the Republic of Korea. The animal protocol was approved by the Institutional Animal Care and Use Committee of Chungbuk National University (Permit Numbers: CBNUA-028-0901-01 and CBNUA-040-0902-01). All rabbits were housed in the animal facility located at the Chungbuk National University Medical School. We made our best efforts to minimize animal suffering in the course of our study. At the terminal stage, the rabbits were anesthetized with intramuscular injections of xylazine (7–10 mg/kg) and ketamine (40–50 mg/kg), and a volume of blood was then collected by intracardiac puncture once per animal, which caused death via exsanguination. In post-mortem examination, we confirmed that the heart had stopped; if it was still beating, then the animal was euthanized with an intracardiac injection of barbiturate euthanasia solution (100 mg/ml).

### Immunoblot analysis

Cells (3×10^5^) were lysed directly with 200 μl of 1× sample loading buffer (80 mM Tris-HCl pH 6.8, 2.0% SDS, 10% glycerol, 0.1 M dithiothreitol, and 0.2% bromophenol blue). Equal amounts of total cell lysates were boiled for 5 min, separated by the Glycine- or the Tricine-SDS-PAGE system, and then transferred to a polyvinylidene difluoride membrane by using a Trans-Blot SD electrophoretic transfer cell (Bio-Rad, Hercules, CA). The blotted membrane was blocked for 1 h at room temperature (RT) with 5% nonfat dried milk in wash buffer (PBS containing 0.2% Tween 20). After three washes (10 min/wash), the blocked membrane was probed for 2 h at RT in wash buffer containing 0.5% nonfat dried milk and one of the following 15 JEV antigen-specific rabbit antisera produced in this study: α-C (1:3,000 dilution), α-pr (1:4,000), α-M (1:1,000), α-E^N-term^ (1:4,000), α-E^C-term^ (1:100), α-NS1^N-term^ (1:3,000), α-NS1'^FS^ (1:1,000), α-NS2A (1:2,000), α-NS2B^Peptide^ (1:1,000), α-NS3^N-term^ (1:1,000), α-NS3^C-term^ (1:1,000), α-NS4A (1:1,000), α-NS4B (1:500), α-NS5^N-term^ (1:1,000), and α-NS5^C-term^ (1:1,000). The primary antibody-decorated membrane was then washed three times (10 min/wash) and incubated for 2 h at RT in wash buffer containing 0.5% nonfat dried milk and a secondary alkaline phosphatase (AP)-conjugated goat α-rabbit IgG (1:5,000; Jackson ImmunoResearch, West Grove, PA). The membrane was washed three times (10 min/wash) and finally developed by incubation with a mixture of 5-bromo-4-chloro-3-indolyl phosphate (BCIP) and nitroblue tetrazolium (NBT) for colorimetric detection of the protein(s) of interest.

### Immunofluorescence assay (IFA)

Cells (1×10^5^) were washed once with PBS and fixed/permeabilized using two different methods: (***i***) fixation with 4% paraformaldehyde in PBS for 10 min at RT, washing twice (10 min/wash) with 0.1 M glycine in PBS, and permeabilization with 0.2% Triton X-100 in PBS for 10 min at RT; and (***ii***) fixation with 100% ice-cold methanol for 10 min at -20°C. In both cases, the fixed/permeabilized cells were washed three times (10 min/wash) with PBS and blocked for 30 min at RT with 5% bovine serum albumin (BSA) in PBS. They were then washed three times (10 min/wash) with PBS and incubated for 1 h at RT in PBS containing 2.5% BSA plus one of our 15 JEV antigen-specific polyclonal rabbit antisera. The primary antibody-treated cells were then washed three times (10 min/wash) with PBS, followed by incubation for 1 h at RT in PBS containing 2.5% BSA plus a Cy3-conjugated goat α-rabbit IgG (1:1,000; Molecular Probes, Eugene, OR). After three washes (10 min/wash) in PBS, the cells were counterstained with 300 nM 4',6-diamidino-2-phenylindole (DAPI, Molecular Probes) in PBS for 5 min at RT. The cells were washed twice (10 min/wash) with PBS and then mounted using an antifade fluorescence mounting medium (Dako, Glostrup, Denmark). Cells were observed under the LSM-710 confocal microscope with a 63× objective (Carl Zeiss, Jena, Germany). Image capture, analysis, and processing were performed using Zen 2010 software (Carl Zeiss).

## Results

### Cloning of 16 small, relatively non-hydrophobic regions of the JEV polyprotein into a bacterial expression vector with the GST affinity tag

**Fig 1:**
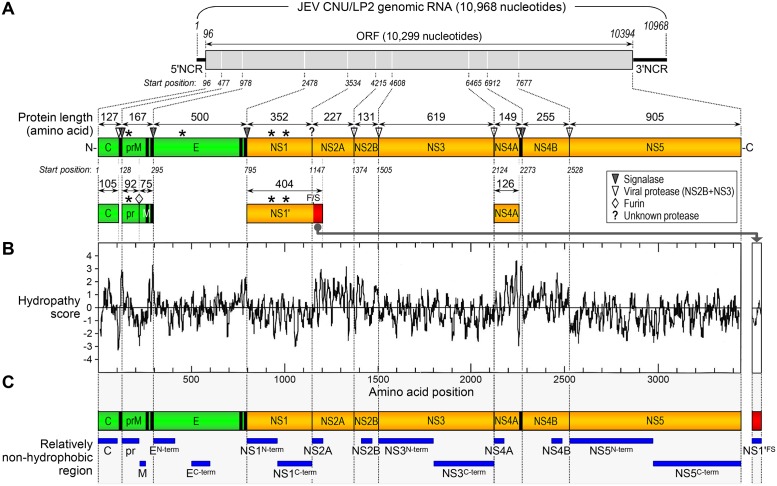
Selection of 16 relatively non-hydrophobic regions located in the viral protein-coding sequences on JEV genomic RNA. (**A**) JEV genome organization and gene expression. The plus-strand genomic RNA of JEV CNU/LP2 consists of a 5'NCR, a single long ORF, and a 3'NCR (top panel). The viral ORF encodes a 3,432-amino acid polyprotein, which is co- or post-translationally processed by host- and virus-encoded proteases into three structural (green) and at least seven nonstructural (orange) proteins (bottom panel). Also, the bottom panel indicates the putative cleavage sites that are conserved among flaviviruses and the length of cleavage products. Vertical black bars represent one or two transmembrane domains located at the C-termini of three structural proteins (C, prM, and E) and at the junction of NS4A/NS4B. Asterisks indicate four potential N-linked glycosylation sites found in the pr portion of prM (Asn-15), E (Asn-154), and NS1/NS1' (Asn-130 and Asn-207). prM is further cleaved by furin into the pr and M proteins during virus assembly and maturation. NS1' is the product of a -1 ribosomal frameshift (F/S) event that occurs between codons 8 and 9 of NS2A, generating the C-terminal extension that includes the N-terminal nine amino acids of NS2A followed by the unique post-frameshift 43 amino acids (red). (**B**) Hydrophobicity plot of the complete 3,432-amino acid polyprotein of JEV CNU/LP2, along with the frameshift-generated 43-amino acid C-terminal extension of NS1'. The average hydropathy scores were calculated using the Kyte-Doolittle method, and regions with scores above 0 are considered hydrophobic. (**C**) Schematic diagram of the 16 non-hydrophobic stretches (horizontal blue bars) predicted in the viral protein-coding regions of JEV CNU/LP2.

We aimed to develop a large panel of polyclonal antisera capable of detecting all of 10 individual viral proteins, using recombinant GST fusion proteins as immunogens. As the viral genetic backbone of choice for GST fusion protein expression, we used JEV CNU/LP2, a highly virulent strain [99] that has been fully sequenced (GenBank accession number AY585243) and cloned as a full-length infectious cDNA [98]. The genomic RNA of JEV CNU/LP2 is 10,968 nucleotides in length, consisting of a 95-nucleotide 5'NCR, a 10,299-nucleotide ORF, and a 574-nucleotide 3'NCR (Fig 1A, top). According to our current understanding of flavivirus gene expression [25,61], the single ORF is translated into a 3,432-amino acid polyprotein, which is proteolytically processed into at least 10 mature proteins (Fig 1A, bottom): three structural (C; prM, a precursor of M; and E) and seven nonstructural (NS1, NS2A, NS2B, NS3, NS4A, NS4B, and NS5) proteins. In WNV, a larger form of NS1 (NS1') has also been reported to be expressed by -1 ribosomal frameshifting at codons 8–9 of NS2A [79,80], adding a total of 52 extra amino acids to the C-terminus of NS1, including the N-terminal 9 amino acids of NS2A and the unique post-frameshift 43 amino acids (Fig 1A, bottom).

**Fig 2:**
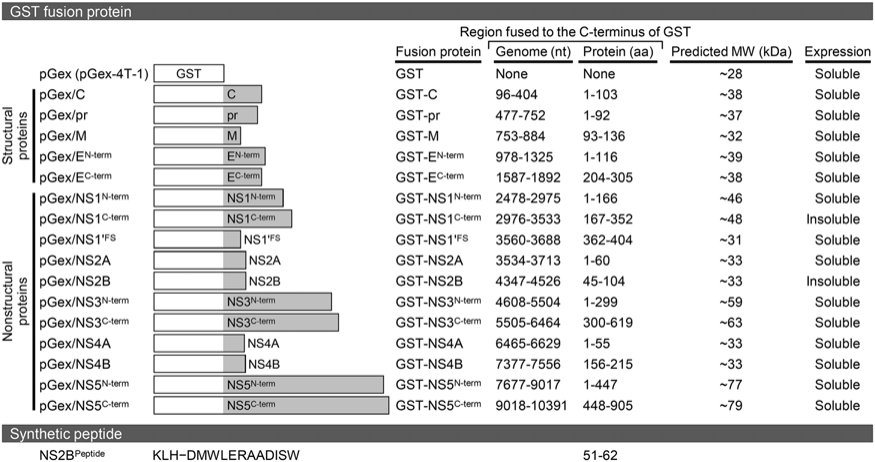
Illustration of 16 GST-tagged recombinant proteins and a KLH-conjugated synthetic oligopeptide. The top panel provides an overview of the 16 GST fusion protein expression constructs. In each construct, a non-hydrophobic region defined in the JEV protein-coding sequences was fused to the C-terminus of GST. The nucleotide (nt) and amino acid (aa) numberings are based on the full-length genome sequence of JEV CNU/LP2 (GenBank accession number AY585243). The predicted molecular weights (MW) are indicated in kDa. Of the 16 GST fusion proteins, while 14 were expressed as a soluble form in *E*. *coli*, the remaining two were produced as an insoluble form. The bottom panel shows a KLH-conjugated synthetic peptide corresponding to amino acids 51–62 of NS2B. Amino acid sequences are abbreviated using the single-letter code.

We first generated the hydrophobicity profile of the complete polyprotein of JEV CNU/LP2, by performing hydropathy analysis using the method of Kyte and Doolittle [100]. The polyprotein hydropathy plot revealed the existence of multiple hydrophobic stretches corresponding to (***i***) one or two transmembrane helices found at the C-termini of the three structural proteins (C, one; prM, two; and E, two) and (***ii***) multiple potential transmembrane domains present within the four nonstructural proteins, NS2A, NS2B, NS4A, and NS4B (Fig 1B). These data agree with the current membrane topology model of the flavivirus polyprotein [101]. In the case of the frameshift product NS1', no specific region was predicted to be highly hydrophobic (Fig 1B). Based on our hydrophobicity profile, we defined a total of 16 unique, relatively non-hydrophobic regions in the entire viral polyprotein and frameshift-generated 43-amino acid C-terminal region of NS1', in order to express these protein fragments in *E*. *coli* as GST-tagged fusion proteins (Fig 1C): one in C, two in prM (designated pr and M), two in E (E^N-term^ and E^C-term^), two in NS1 (NS1^N-term^ and NS1^C-term^), one in NS1' (NS1'^FS^), one in NS2A, one in NS2B, two in NS3 (NS3^N-term^ and NS3^C-term^), one in NS4A, one in NS4B, and two in NS5 (NS5^N-term^ and NS5^C-term^). Each of these 16 selected coding regions was PCR-amplified from the full-length infectious cDNA of JEV CNU/LP2 and cloned into the bacterial expression vector pGex-4T-1 to yield in-frame fusion proteins with an N-terminal GST tag (Fig 2, GST fusion protein).

### Expression and purification of soluble GST-tagged recombinant proteins

To determine whether the 16 JEV cDNA fragments cloned into pGex-4T-1 are expressed as soluble GST fusion proteins after IPTG induction in *E*. *coli* BL21, we compared the soluble (supernatant) and insoluble (pellet) fractions of the sonicated total bacterial lysates by SDS-PAGE. Of the 16 preparations, we found that 14 GST fusion proteins of the expected sizes were detected predominantly in the soluble fraction; the remaining two (GST-NS1^C-term^ and GST-NS2B), although having the predicted sizes, were found mostly in the insoluble fraction (summarized in Fig 2, GST fusion protein). For these last two fusion proteins, we made multiple attempts to circumvent the insolubility of the induced proteins by testing protein expression at three different temperatures (25°C, 30°C, and 35°C) for 1 to 4 h, but observed only minor effects of temperature on their solubility. Thus, the 14 soluble GST-tagged fusion proteins, excluding GST-NS1^C-term^ and GST-NS2B, were purified from the pre-cleared bacterial lysates by glutathione-affinity chromatography. Subsequently, each of the purified GST fusion proteins was analyzed by SDS-PAGE in a 10 or 12% gel, as appropriate, in parallel and under the same experimental conditions with the free GST protein purified from *E*. *coli* that had been transformed with pGex-4T-1 without any insert.

**Fig 3:**
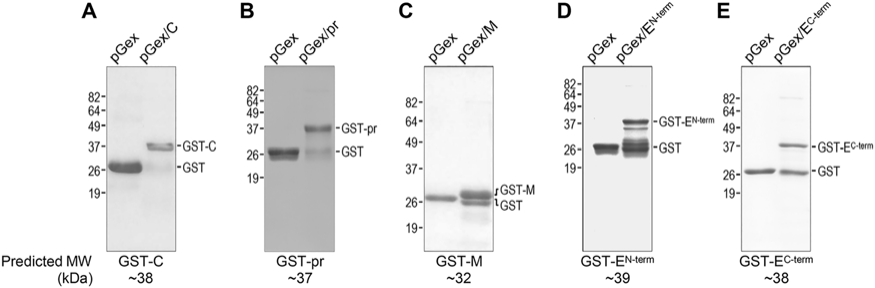
Production of five GST fusion proteins, each containing a non-hydrophobic region from the JEV structural protein-coding region. GST fusion proteins were expressed from pGex-4T-1 vector in *E*. *coli* and purified from bacterial lysates by affinity chromatography using glutathione-Sepharose. About 5–10 μg of each purified protein was resolved on a SDS-polyacrylamide gel and stained with Coomassie blue. The five GST fusion proteins we generated are: GST-C (**A**), GST-pr (**B**), GST-M (**C**), GST-E^N-term^ (**D**), and GST-E^C-term^ (**E**). Free GST protein was used as control. Molecular weight markers are indicated in kDa on the left side of each panel, and GST and GST fusion proteins are marked on the right side. The predicted molecular weights are indicated at the bottom of each panel.

**Fig 4:**
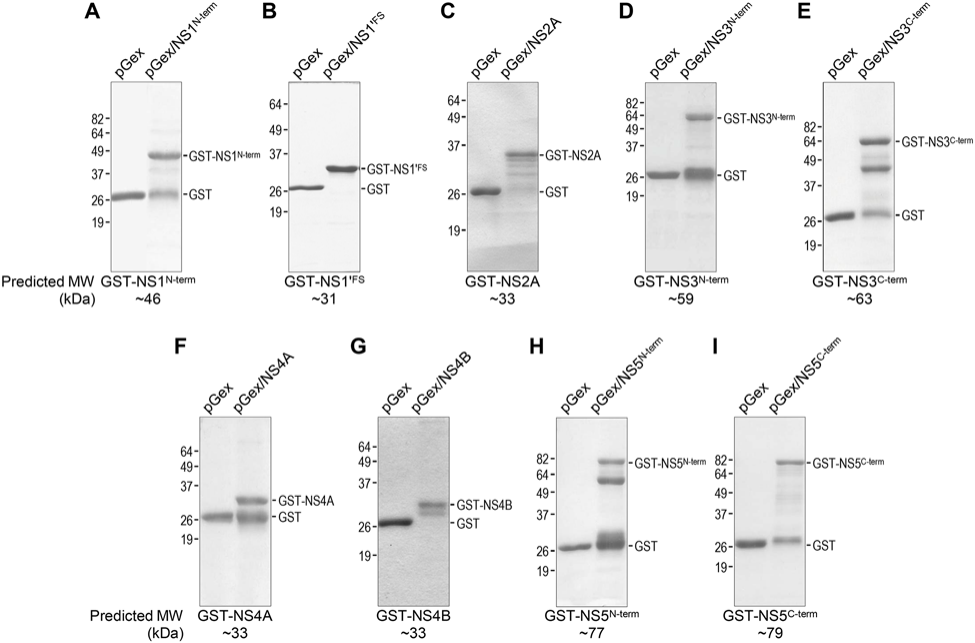
Production of nine GST fusion proteins, each containing a non-hydrophobic region from the JEV nonstructural protein-coding region. GST fusion proteins were prepared and analyzed as described in the legend of Fig 3. The nine GST fusion proteins we produced are: GST-NS1^N-term^ (**A**), GST-NS1'^FS^ (**B**), GST-NS2A (**C**), GST-NS3^N-term^ (**D**), GST-NS3^C-term^ (**E**), GST-NS4A (**F**), GST-NS4B (**G**), GST-NS5^N-term^ (**H**), and GST-NS5^C-term^ (**I**). All parts of the figure are labeled as in Fig 3.

Fig 3 shows the results of SDS-PAGE analysis of the five purified GST fusion proteins, each containing a non-hydrophobic region found in one of the viral structural proteins. Fig 4 presents those of the nine purified GST fusion proteins, each containing a non-hydrophobic region located in one of the viral nonstructural proteins. In all 14 cases, expression of the individual GST fusion proteins was only observed in bacterial cells transformed with the corresponding recombinant pGex constructs, and not with the empty vector. In SDS-PAGE gels, the expression patterns of these purified GST fusion proteins could be grouped into four classes: (***i***) Class I (three cases), appearing predominantly as a single band corresponding in size to the respective full-length fusion protein (GST-C, Fig 3A; GST-pr, Fig 3B; and GST-NS1'^FS^, Fig 4B); (***ii***) Class II (two cases), which migrated as a major band of the expected molecular weight, with one to three minor bands larger than that of the ~28-kDa GST (GST-NS2A, Fig 4C and GST-NS4B, Fig 4G); (***iii***) Class III (six cases), detected as two discrete bands corresponding to the full-length fusion protein and to the free GST (GST-M, Fig 3C; GST-E^C-term^, Fig 3E; GST-NS1^N-term^, Fig 4A; GST-NS3^N-term^, Fig 4D; GST-NS4A, Fig 4F; and GST-NS5^C-term^, Fig 4I); and (***iv***) Class IV (three cases), similar to class III but with at least one additional discrete band corresponding to the truncated form(s) of the respective full-length fusion proteins (GST-E^N-term^, Fig 3D; GST-NS3^C-term^, Fig 4E; and GST-NS5^N-term^, Fig 4H). We confirmed by immunoblotting that all 14 full-length GST fusion proteins and their truncated forms reacted with an α-GST rabbit antiserum (data not shown). Thus, our results showed that, except for NS2B, we successfully expressed and purified one or two non-hydrophobic polypeptide segments, located in each of the 10 major viral proteins, with the N-terminal GST tag.

### Generation of 15 JEV antigen-specific polyclonal antisera in rabbits

For the production of antisera, we chose rabbits because of their convenient body size, ease of handling, relatively long lifespan, and ample volume of antiserum available. Immunization was performed on a pair of rabbits for each antigen, using the following 14 recombinant GST fusion proteins described above: GST-C, GST-pr, GST-M, GST-E^N-term^, GST-E^C-term^, GST-NS1^N-term^, GST-NS1'^FS^, GST-NS2A, GST-NS3^N-term^, GST-NS3^C-term^, GST-NS4A, GST-NS4B, GST-NS5^N-term^, and GST-NS5^C-term^. These 14 fusion proteins cover all the 10 major JEV proteins except NS2B. To raise antisera against NS2B, we immunized two rabbits with a KLH-conjugated synthetic oligopeptide (NS2B^Peptide^) corresponding to amino acids 51–62 of NS2B (Fig 2, Synthetic peptide). Two weeks after the initial immunization, the rabbits were boosted once a week for a maximum of 5 weeks with the same fusion protein or synthetic peptide antigen and were test-bled 5 days after the third boost. Serum samples were tested by immunoblotting to monitor the presence of antibodies capable of recognizing the respective immunogens. In all 15 cases, a detectable level of antibody was observed in the serum of at least one of the two immunized rabbits after the third boost (data not shown), and all the immune sera were therefore collected 5 days after the fifth boost. We therefore obtained positive sera from at least one animal per antigen.

### Profiling of all the viral proteins produced in JEV-infected cells by immunoblotting

Using our 15 JEV region-specific rabbit antisera, we aimed to profile all the viral proteins expressed in JEV-infected cells. To achieve our goal, subconfluent monolayers of JEV-susceptible BHK-21 cells were mock-infected or infected with JEV CNU/LP2 at an MOI of 1. At 18 h post-infection (hpi), cell monolayers were lysed directly with SDS-based lysis buffer, and equal amounts of total cell lysates were separated by SDS-PAGE under reducing conditions and analyzed by immunoblotting.

**Fig 5:**
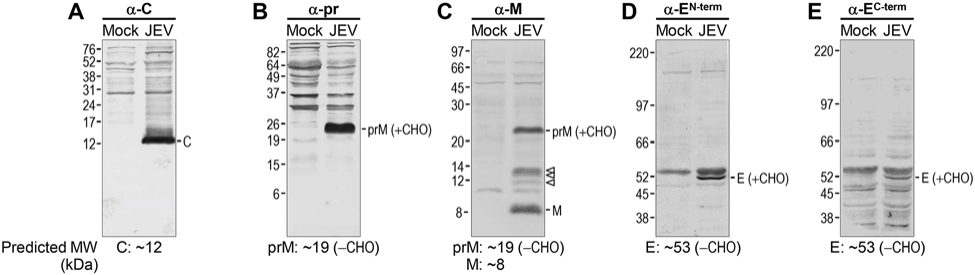
Identification of viral structural proteins in JEV-infected cells by immunoblotting. Naïve BHK-21 cells were mock-infected or infected with JEV CNU/LP2 at an MOI of 1. At 18 hpi, equal amounts of whole cell lysates from mock-infected (Mock) and JEV-infected (JEV) cells were analyzed by immunoblotting with each of the following five primary antibodies: α-C (**A**), α-pr (**B**), α-M (**C**), α-E^N-term^ (**D**), and α-E^C-term^ (**E**). The immunoreactive proteins were visualized by probing with a secondary AP-conjugated goat α-rabbit IgG, followed by enzymatic reaction of AP with a mixture of BCIP and NBT. Molecular size markers in kDa are shown on the left of each blot, and the three viral structural proteins (C, prM/M, and E) are indicated on the right. Also noted are three additional α-M-reactive proteins (open arrowhead). Each prM and E possesses one potential N-linked glycosylation site (CHO). The predicted molecular weights of the respective viral structural proteins are provided at the bottom of each panel.

Fig 5 shows the results of five immunoblots; each reacted with an antiserum specific to a defined region in one of the three viral structural proteins, GST-C (α-C), GST-pr (α-pr), GST-M (α-M), GST-E^N-term^ (α-E^N-term^), and GST-E^C-term^ (α-E^C-term^). We found that (***i***) α-C recognized a distinct protein band of ~12 kDa (Fig 5A), the predicted size of the 105-amino acid C protein that lacks its C-terminal transmembrane domain [102–104]. (***ii***) α-pr detected a species with a molecular weight of ~24 kDa (Fig 5B), the prM precursor that contains an N-linked glycan at Asn-15 in its pr domain [99]. (***iii***) α-M reacted strongly with two major proteins, the precursor prM (~24 kDa) and its cleavage product M (~8 kDa) (Fig 5C). It also reacted weakly with three minor proteins of molecular weights ranging from ~10 to 14 kDa (Fig 5C, open arrowhead). (***iv***) Both α-E^N-term^ and α-E^C-term^ revealed a major protein band at ~53 kDa, corresponding to the predicted size of the nascent E protein (Fig 5D and 5E). On the gel, however, the E protein was compressed and pushed down by a large amount of cellular proteins migrating just above it, causing a tendency for it to run faster than its actual molecular weight. This observation is noteworthy, since the E protein has a single potential N-linked glycosylation site at Asn-154. An alternative explanation for the apparent molecular weight is the lack of glycosylation. In all cases, control experiments using pre-immune sera from the same rabbits did not show any signal in immunoblot analysis (data not shown).

**Fig 6:**
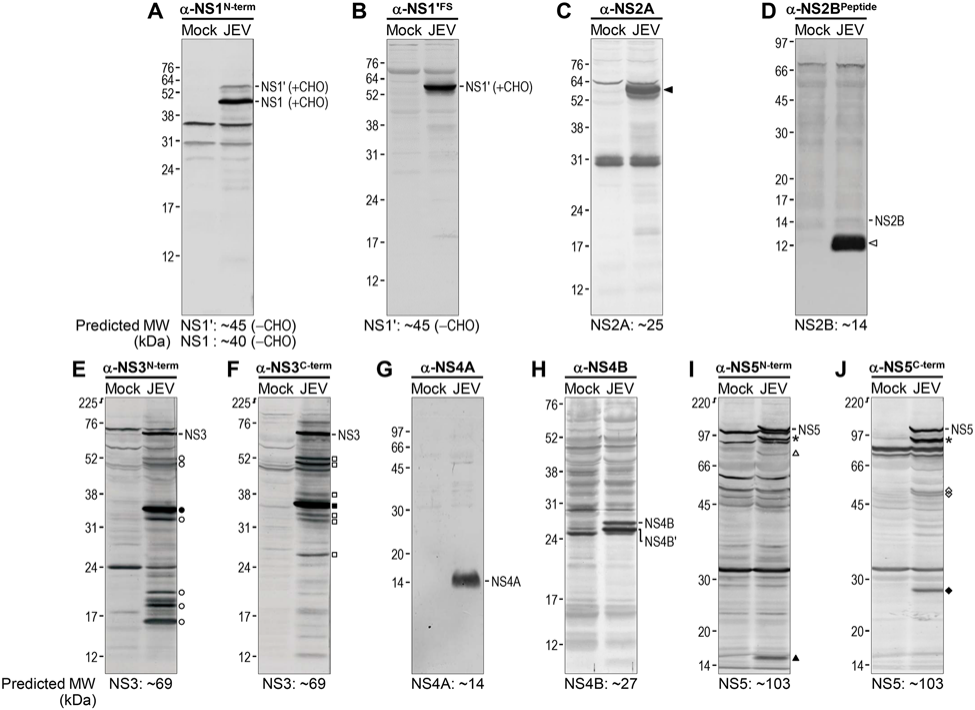
Identification of viral nonstructural proteins in JEV-infected cells by immunoblotting. Total cell lysates from mock-infected (Mock) and JEV-infected (JEV) cells were prepared as described in Fig 5 and examined by immunoblotting with each of the following 10 primary antibodies: α-NS1^N-term^ (**A**), α-NS1'^FS^ (**B**), α-NS2A (**C**), α-NS2B^Peptide^ (**D**), α-NS3^N-term^ (**E**), α-NS3^C-term^ (**F**), α-NS4A (**G**), α-NS4B (**H**), α-NS5^N-term^ (**I**), and α-NS5^C-term^ (**J**). The immunoreactive proteins were detected by probing with a secondary AP-conjugated goat α-rabbit IgG and visualized by reaction with a mixture of BCIP and NBT. All parts of the figure are labeled as in Fig 5. Highlighted are a significant number of viral proteins that reacted with the given antiserum (see the main text for more details on the individual immunoreactive proteins). NS1 and NS1' share two potential N-linked glycosylation sites (CHO).

Fig 6 shows the results of 10 immunoblots, each probed with an antiserum specific to a defined portion in one of the eight viral nonstructural proteins, including NS1': GST-NS1^N-term^ (α-NS1^N-term^), GST-NS1'^FS^ (α-NS1'^FS^), GST-NS2A (α-NS2A), NS2B^Peptide^ (α-NS2B^Peptide^), GST-NS3^N-term^ (α-NS3^N-term^), GST-NS3^C-term^ (α-NS3^C-term^), GST-NS4A (α-NS4A), GST-NS4B (α-NS4B), GST-NS5^N-term^ (α-NS5^N-term^), and GST-NS5^C-term^ (α-NS5^C-term^). We noted that (***i***) α-NS1^N-term^ recognized two prominent protein bands, one major NS1 (~45 kDa) and the other minor NS1' (~58 kDa), both of which are ~5- to 13-kDa larger than predicted by their amino acid sequences (Fig 6A). The observed increases in molecular weight are consistent with the presence of two potential N-linked glycosylation sites (Asn-130 and Asn-207) in the NS1 protein-coding region that it also shares with the frameshift product NS1' [79,80,105]. (***ii***) α-NS1'^FS^ only reacted strongly with the predicted ~58-kDa NS1' (Fig 6B). (***iii***) α-NS2A revealed an unexpected cluster of multiple protein bands at ~58 kDa (Fig 6C, closed arrowhead), with no convincing appearance of the predicted ~25-kDa NS2A (for more detail, see below under “Immunoreactivity of the α-NS2A antiserum”). (***iv***) α-NS2B^Peptide^ detected an unexpected but intensely stained protein band at ~12 kDa (Fig 6D, open arrowhead), with the expected but barely detectable ~14-kDa NS2B (Fig 6D). (***v***) Both α-NS3^N-term^ and α-NS3^C-term^ recognized equally well the predicted ~69-kDa NS3 (Fig 6E and 6F). Intriguingly, each of these antisera also reacted more strongly with another major protein of ~34–35 kDa (Fig 6E, closed circle and Fig 6F, closed square), and less intensely with multiple minor proteins in the range of ~16–52 kDa (Fig 6E, open circle and Fig 6F, open square; for more detail, see below under “Identification of multiple NS3- and NS5-related proteins in JEV-infected cells”). (***vi***) α-NS4A stained a single protein band of ~14 kDa (Fig 6G), the predicted size of the 126-amino acid NS4A protein that lacks its C-terminal transmembrane region [106,107]. (***vii***) α-NS4B recognized a doublet of ~25-kDa and ~27-kDa proteins (Fig 6H). Of these two, the slow-migrating band corresponds to the predicted size of NS4B, and the fast-migrating band (NS4B') is suggested to be generated by an unknown post-translational modification [107,108]. (***viii***) Both α-NS5^N-term^ and α-NS5^C-term^ probed the predicted ~103-kDa NS5 (Fig 6I and 6J) and another ~90-kDa protein (Fig 6I and 6J, asterisk) equally well. Also, each of these antisera reacted strongly with an additional protein of ~15 kDa (Fig 6I, closed triangle) or ~27 kDa (Fig 6J, closed diamond), and to some extent, with one to two minor proteins of ~50–75 kDa (Fig 6I, open triangle and Fig 6J, open diamond; for more detail, see below under “Identification of multiple NS3- and NS5-related proteins in JEV-infected cells”). Control pre-immune sera from the same animals were all negative in immunoblot analysis (data not shown).

### Immunoreactivity of the α-NS2A antiserum

**Fig 7:**
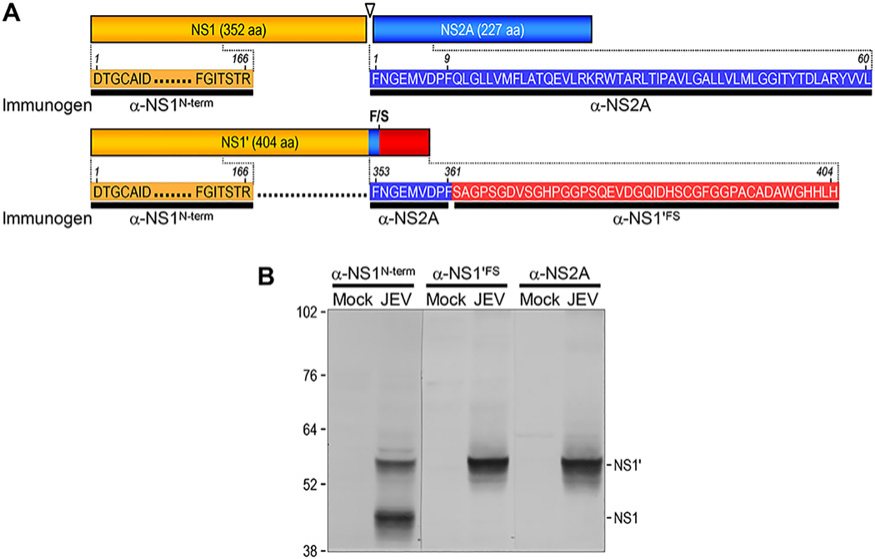
Size comparison of NS1' with the α-NS2A-reactive protein. (**A**) Schematic diagram showing the location of the immunogens of three antisera: α-NS1^N-term^ (orange), α-NS1'^FS^ (red), and α-NS2A (blue). Highlighted is the overlapping 9-amino acid immunogen of α-NS2A, just upstream of the ribosomal frameshift (F/S) site for the expression of NS1'. An open arrowhead indicates the putative NS1-NS2A cleavage site. aa, amino acids. (**B**) Direct comparison of the electrophoretic mobility between the α-NS1'^FS^-recognized NS1' and the α-NS2A-reactive protein. Naïve BHK-21 cells were mock-infected or infected with JEV CNU/LP2 at an MOI of 1 for 18 h. Three cell lysate pairs made from mock-infected (Mock) and JEV-infected (JEV) cells were run in parallel on a single SDS-polyacrylamide gel. The fractionated proteins were transferred onto a PVDF membrane, which was then divided into three equal parts. Each blot was immunoblotted individually using α-NS1^N-term^, α-NS1'^FS^, and α-NS2A. The positions of molecular weight markers (kDa) are shown on the left of the figure, and NS1 and NS1' are indicated on the right.

In denaturing SDS-PAGE separations, we noted that the α-NS2A-reactive ~58-kDa protein was similar in size to the NS1' protein recognized by both α-NS1^N-term^ and α-NS1'^FS^ (Fig 6A–6C). According to the previous studies with WNV, NS1' is the product of a -1 ribosomal frameshift event that occurs at codons 8–9 of NS2A [79,80]; it is thus synthesized as a 404-amino acid transframed fusion protein including the complete 352 amino acids of NS1, the N-terminal nine amino acids of NS2A, and the post-frameshift 43-amino acid peptide (Fig 7A). Given that α-NS2A was directed against the N-terminal 60 amino acids of NS2A, it is likely that the antiserum reacts with the internal overlapping 9-amino acid immunogen, amino acids 353 through 361, of NS1' (Fig 7A). To directly compare their migration rates on the same gel, we separated triplicate sets of the total cell lysates from mock- and JEV-infected BHK-21 cells by denaturing SDS-PAGE in a large gel format (16 x 20 cm) for high resolution and transferred the proteins onto a single blotting membrane. The membrane was then cut into three equal blots, each of which was individually probed with α-NS1^N-term^, α-NS1'^FS^, and α-NS2A. Indeed, we found that the α-NS2A-reactive ~58-kDa protein precisely co-migrated with the NS1' protein (Fig 7B). These results support the notion that α-NS2A is capable of detecting the NS1' protein, which shares the N-terminal nine amino acids with NS2A; however, there is still a possibility that the α-NS2A-reactive protein might be an unrelated protein co-migrating coincidentally with the NS1' protein.

### Identification of multiple NS3- and NS5-related proteins in JEV-infected cells

**Fig 8:**
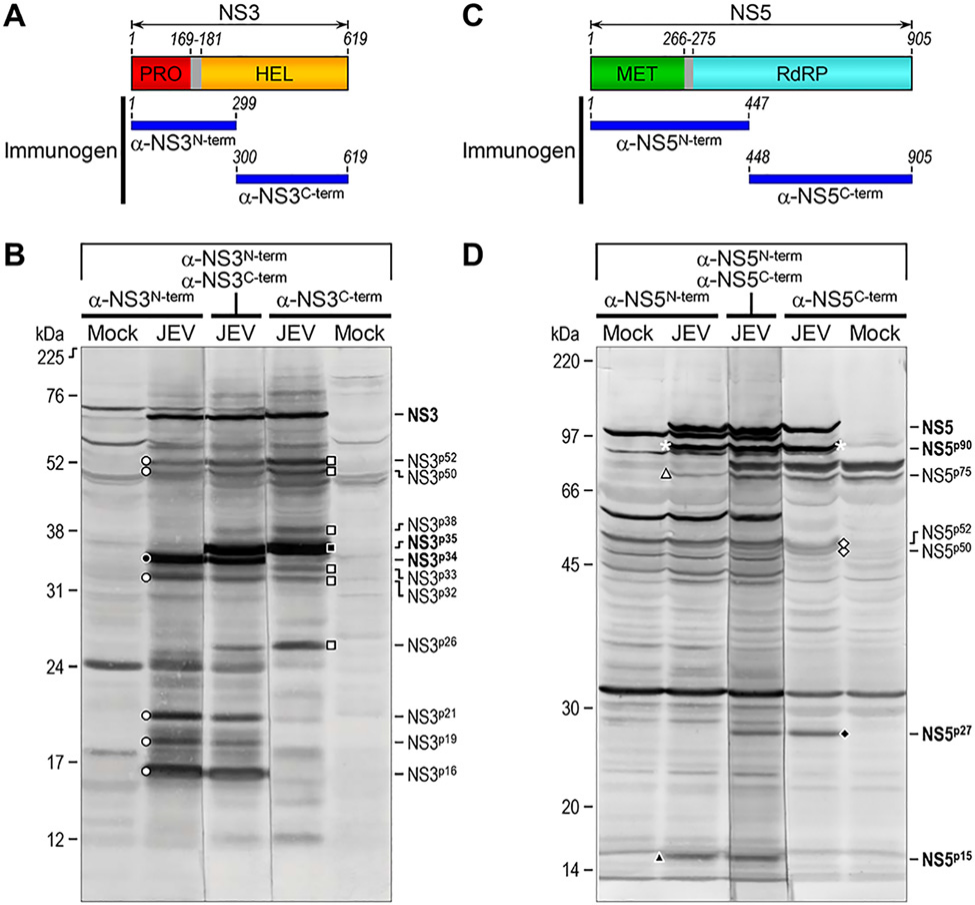
Size comparison of multiple NS3- and NS5-related proteins accumulated in JEV-infected cells. (**A**) Schematic diagram of the full-length NS3 protein composed of an N-terminal protease domain (PRO, red), a short interdomain linker (residues 169–181, gray), and a C-terminal RNA helicase/NTPase/RTPase domain (HEL, orange). Indicated below the full-length NS3 diagram are the two immunogens for α-NS3^N-term^ and α-NS3^C-term^ (horizontal blue bar). (**B**) Immunoblot analysis of lysates from BHK-21 cells mock-infected or infected with JEV CNU/LP2 at an MOI of 1. At 18 hpi, whole cell lysates were harvested for immunoblotting with α-NS3^N-term^ or α-NS3^C-term^ individually, or both simultaneously. Predominant immunoreactive NS3-related proteins are marked by the same symbols as in Fig 6E and 6F and named according to their approximate molecular weights. (**C**) Schematic diagram of the full-length NS5 protein containing an N-terminal MTase/GTase domain (MET, green), a short interdomain linker (residues 266–275, gray), and a C-terminal RNA-dependent RNA polymerase domain (RdRP, cyan). Labeled below the full-length NS5 diagram are the two immunogens for α-NS5^N-term^ and α-NS5^C-term^ (horizontal blue bar). (**D**) Immunoblot analysis of lysates from BHK-21 cells mock-infected or infected with JEV CNU/LP2 at an MOI of 1. At 18 hpi, total cell lysates were harvested for immunoblotting with α-NS5^N-term^ or α-NS5^C-term^ individually, or both simultaneously. Major immunoreactive NS5-related proteins are marked by the same symbols as in Fig 6I and 6J and named according to their approximate molecular weights. Molecular weight markers in kDa are shown on the left of each figure, and multiple NS3- and NS5-related proteins are indicated on the right.

In the case of both NS3 and NS5, in JEV-infected BHK-21 cells we detected not only the expected full-length proteins, but also a number of unexpected smaller proteins that reacted with either one or both of the rabbit antisera directed against the N- or C-terminal half of one of the two viral proteins, i.e., α-NS3^N-term^/α-NS3^C-term^ (Fig 6E and 6F) and α-NS5^N-term^/α-NS5^C-term^ (Fig 6I and 6J). For each protein, we sought to specify the reactivity of its antiserum pair and identify the immunoreactive proteins uniquely recognized by the N- or C-terminal specific antiserum on immunoblots. With that goal in mind, we electrophoresed two identical sets of the total cell lysates from mock- and JEV-infected BHK-21 cells side-by-side in an SDS-PAGE gel and then transferred them to a single blotting membrane. The membrane was split into two equal blots, which were individually probed with either the N- or C-terminal-specific antiserum. In parallel, an aliquot of the same JEV-infected cell lysate was also included in between the two sample sets, and the corresponding membrane strip was stained with both the N- and C-terminal specific antisera to serve as a reference for all the immunoreactive proteins (Fig 8).

NS3 contains an N-terminal serine protease domain (PRO) connected to a C-terminal RNA helicase/NTPase/RTPase domain (HEL) by a 13-residue interdomain linker (residues 169–181) [101,109]. With respect to the antigens recognized by the two NS3-specific antisera, α-NS3^N-term^ was directed against the N-terminal 299 residues of NS3 that constitute the entire PRO, linker, and N-terminal 118 residues of HEL; on the other hand, α-NS3^C-term^ was raised against the remaining C-terminal 320 residues of HEL (Fig 8A). Based on their reactivity with either one or both of the α-NS3^N-term^ and α-NS3^C-term^ antisera, we were able to group all 12 major NS3-related proteins into three classes (Fig 8B; note that NS3-related proteins are designated by a superscript “p” followed by the number indicating the approximate molecular weight based on the electrophoretic mobility): (***i***) Class I, comprising four α-NS3^N-term^-positive, α-NS3^C-term^-positive proteins, i.e., the full-length NS3 (~69 kDa) and three others (NS3^p32^, NS3^p50^, and NS3^p52^); (***ii***) Class II, comprising four α-NS3^N-term^-positive, α-NS3^C-term^-negative proteins (NS3^p16^, NS3^p19^, NS3^p21^, and NS3^p34^); and (***iii***) Class III, comprising four α-NS3^N-term^-negative, α-NS3^C-term^-positive proteins (NS3^p26^, NS3^p33^, NS3^p35^, and NS3^p38^). Among these, the two most strongly immunoreactive proteins were NS3^p34^ and NS3^p35^, which reacted specifically with α-NS3^N-term^ and α-NS3^C-term^, respectively (Fig 8B).

NS5 includes an N-terminal MTase/GTase domain (MET) joined to a C-terminal RdRP region by a 10-residue interdomain linker (residues 266–275) [110]. With regard to the antigens recognized by the two NS5-specific antisera, α-NS5^N-term^ was raised against the N-terminal 447 residues of NS5 that represent the entire MET, linker, and N-terminal 172 residues of RdRP; contrarily, α-NS5^C-term^ was directed to the remaining C-terminal 458 residues of RdRP (Fig 8C). Depending on their reactivity with either one or both of the α-NS5^N-term^ and α-NS5^C-term^ antisera, seven primary NS5-related proteins were arranged into four classes (Fig 8D; note that NS5-related proteins are labeled by a superscript “p” followed by the number indicating the approximate molecular weight based on the electrophoretic mobility): (***i***) Class I, represented by two α-NS5^N-term^-positive, α-NS5^C-term^-positive proteins, i.e., the full-length NS5 (~103 kDa) and an ~90-kDa protein (NS5^p90^); (***ii***) Class II, represented by an α-NS5^N-term^-positive, α-NS5^C-term^-negative protein (NS5^p15^); (***iii***) Class III, represented by an α-NS5^N-term^-negative, α-NS5^C-term^-positive protein (NS5^p27^); and (***iv***) Class IV, represented by three proteins (NS5^p50^, NS5^p52^, and NS5^p75^) that were weakly reactive with at least α-NS5^N-term^ or α-NS5^C-term^, but their specificities could not be clearly determined because of a cross-reactivity to cellular proteins. Of these, the full-length NS5 and three others (NS5^p15^, NS5^p27^, and NS5^p90^) were relatively more immunoreactive (Fig 8D).

### Detection of viral proteins in JEV-infected cells by IFA

Using a panel of 15 JEV antigen-specific rabbit antisera, we performed indirect IFAs with confocal microscopy to assess their reactivity and visualize viral proteins in JEV-infected cells. In the initial series of IFA experiments, we compared five fixatives of two different types: cross-linking agents (paraformaldehyde and formaldehyde), which form covalent bonds between proteins and create a network of cross-linked antigens; and organic solvents (methanol, ethanol, and acetone), which remove lipids, precipitate proteins, and dehydrate cells. Our results showed that the IF staining patterns differed between the two fixative types but were nearly identical within the same fixative type (data not shown). We therefore present the results of indirect IFAs for two representative fixatives, paraformaldehyde and methanol.

For analysis by indirect IFA, BHK-21 cells were mock-infected or infected with JEV CNU/LP2 at an MOI of 1 for 18 h. Infected cells were fixed in 4% paraformaldehyde for 10 min and permeabilized with 0.2% Triton X-100 for an additional 10 min. Alternatively, the infected cells were fixed/permeabilized in one step in 100% methanol for 10 min, because this fixative dissolves the lipids of the cell membranes making them permeable to antibodies. In both cases, cells were then processed for indirect IFA by staining with one of the 15 JEV antigen-specific rabbit antisera and a secondary Cy3-conjugated goat α-rabbit IgG. Cell nuclei were counterstained with DAPI. The images were taken by a laser-scanning confocal microscope.

**Fig 9:**
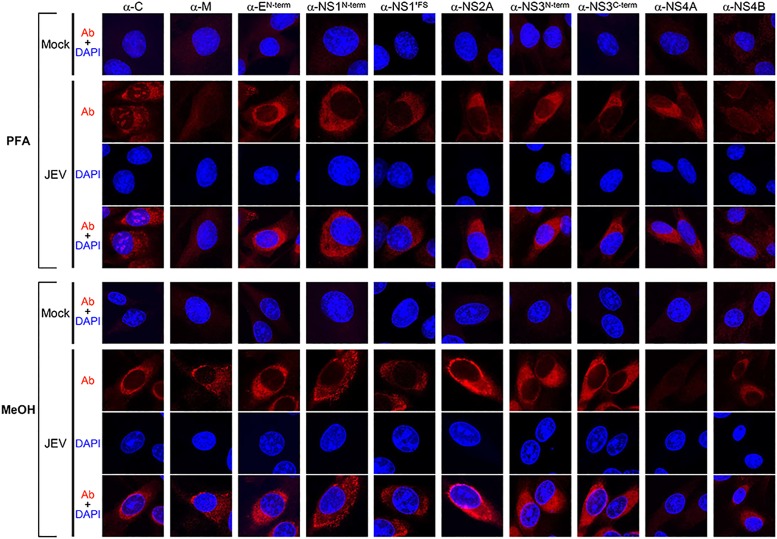
Fluorescence images of viral proteins detected in JEV-infected cells, depending on the method of cell fixation/permeabilization. Naïve BHK-21 cells were mock-infected or infected with JEV CNU/LP2 at an MOI of 1. At 18 hpi, cells were fixed/permeabilized with paraformaldehyde/Triton X-100 (PFA) or methanol (MeOH). Subsequently, cells were analyzed by indirect IFA using one of the following 10 primary antibodies: α-C, α-M, α-E^N-term^, α-NS1^N-term^, α-NS1'^FS^, α-NS2A, α-NS3^N-term^, α-NS3^C-term^, α-NS4A, and α-NS4B. The primary antibody-reactive proteins were stained with a secondary Cy3-conjugated goat α-rabbit IgG (red). The nuclei were counterstained with DAPI (blue). Fluorescence images were taken with a laser scanning confocal microscope with a 63 oil objective lens. Representative unmerged (Ab or DAPI) and merged (Ab+DAPI) images are shown.

Table 2 summarizes the immunoreactivity of all 15 JEV antigen-specific rabbit antisera, with the optimal dilution of each antiserum determined by both paraformaldehyde- and methanol-based fixation. Overall, 10 of the 15 antisera clearly reacted with the corresponding viral antigens after fixation with the cross-linking fixatives and/or organic solvent-based fixatives: i.e., α-C, α-M, α-E^N-term^, α-NS1^N-term^, α-NS1'^FS^, α-NS2A, α-NS3^N-term^, α-NS3^C-term^, α-NS4A, and α-NS4B. Of the remaining five antisera, however, three (α-pr, α-E^C-term^, and α-NS2B^Peptide^) stained neither viral nor cellular proteins, whereas two (α-NS5^N-term^ and α-NS5^C-term^) cross-reacted intensely with cellular proteins (data not shown). Fig 9 shows representative confocal IF images of JEV-infected BHK-21 cells after staining with each of the 10 antisera capable of detecting the corresponding viral proteins. In all 10 cases, a predominant fluorescence signal was invariably accumulated in the perinuclear region of the infected cells, where viral replication takes place. Except for α-NS4A, a more intense fluorescence signal was observed with the organic solvent-based fixatives than with the cross-linking fixatives, as evidenced by the appearance of granule-like foci in the perinuclear area (Fig 9, PFA vs. MeOH). Interestingly, the JEV C protein was concentrated not only in the perinuclear area of JEV-infected cells but also inside their nuclei, and this nuclear localization was exhibited by cross-linking fixation and detergent permeabilization, but not by organic solvent-based fixation/permeabilization (Fig 9, α-C). Taken together, our results demonstrate the reactivity of 15 JEV antigen-specific rabbit antisera by indirect IFAs with confocal microscopy.

**Table 2:** Reactivity of 15 JEV antigen-specific polyclonal antisera by confocal indirect IFA.

		Immunoreactivity (optimal dilution)	
Target protein	Antiserum	PFA^*a*^ fixation	Methanol fixation
C	α-C	Yes (1:2,000)	Yes (1:2,000)
prM (pr)	α-pr	No	No
prM (M)	α-M	No	Yes (1:250)
E	α-E^N-term^	Yes (1:1,000)	Yes (1:2,000)
	α-E^C-term^	No	No
NS1	α-NS1^N-term^	Yes (1:1,000)	Yes (1:2,000)
NS1'	α-NS1'^FS^	Yes (1:2,000)	Yes (1:2,000)
NS2A	α-NS2A	Yes (1:1,000)	Yes (1:2,000)
NS2B	α-NS2B^Peptide^	No	No
NS3	α-NS3^N-term^	Yes (1:1,000)	Yes (1:1,000)
	α-NS3^C-term^	Yes (1:4,000)	Yes (1:4,000)
NS4A	α-NS4A	Yes (1:250)	No
NS4B	α-NS4B	No	Yes (1:100)
NS5	α-NS5^N-term^	SNBS^*b*^	SNBS
	α-NS5^C-term^	SNBS	SNBS

^*a*^ PFA, Paraformaldehyde.

^*b*^ SNBS, Strong non-specific background staining.

## Discussion

In this work, we have presented a comprehensive landscape of viral gene products in susceptible BHK-21 cells infected with a pathogenic wild-type JEV strain CNU/LP2 [98,99,111], as determined by immunoblot and immunofluorescence analyses with a panel of 15 JEV-specific rabbit antisera against all parts of the coding regions in the viral genome. Using these 15 antisera suitable for immunoblot analysis, we identified all the major viral proteins expressed from the viral genomic RNA except the predicted ~25-kDa NS2A. We also demonstrated that 10 of the 15 antisera were applicable to indirect IFA after either cross-linking and/or organic solvent-based fixation and were capable of visualizing the eight major viral proteins primarily localized in the perinuclear region of the JEV-infected cells: C, prM/M, E, NS1, NS1', NS3, NS4A, and NS4B. Our data show the first global picture of JEV gene expression in infected cells, allowing us to detect several JEV gene products that had not been previously identified.

When tested with lysates of JEV-infected BHK-21 cells by immunoblotting, a set of five rabbit antisera against the JEV structural proteins reacted well with the corresponding viral proteins (C, prM/M, and E) at their predicted molecular weights, in good agreement with the findings of previous studies of JEV [112] and other flaviviruses [62,102–104,108,113–115]. In addition to prM (~24 kDa) and its cleavage product M (~8 kDa), three additional immunoreactive proteins of ~10–14 kDa were detected with α-M, but not with α-pr, suggesting that multiple non-canonical endoproteolytic cleavages occur in the pr portion of prM, upstream of the canonical pr-M cleavage site. Our finding is intriguing because recent studies with DENV have shown that an incomplete cleavage of prM generates a high proportion of partially mature prM/M-containing particles, and the ratio of prM to M determines the viral infectivity [116,117]. Also, it has been demonstrated that completely immature prM-containing DENV particles are non-infectious [118,119] but become infectious upon interaction with α-prM antibodies, because the interacting antibodies promote the cell entry of immature particles into Fc receptor-expressing cells [120]. Furthermore, the cysteine-induced structural perturbation of prM has been found to be capable of restoring the infectivity of a furin cleavage-deficient mutant TBEV, by circumventing the need for intracellular furin-mediated cleavage [121]. Thus, it will be interesting to determine whether the multiple non-canonical endoproteolytic cleavages of JEV prM take place in other susceptible cells, as observed in BHK-21 cells, and whether they are mediated by furin or a furin-like host cell protease, as demonstrated for the canonical pr-M cleavage [95]. Further studies are needed to assess the biological significance of the multiple non-canonical cleavages of JEV prM for viral replication and pathogenesis.

Despite its role as a structural protein, the C protein of flaviviruses has been shown to be localized not only to the cytoplasm but also to the nuclei of infected cells, when the cells are fixed/permeabilized with methanol, acetone, formalin, paraformaldehyde/Triton X-100, or formaldehyde/methanol [122–128]. Consistent with these previous studies, we also observed by indirect IFA with confocal microscopy that JEV C was localized to both the cytoplasm and nucleus of JEV-infected BHK-21 cells. It is worthy of note, however, that detection of the protein in the nuclei was dependent on the cell fixation/permeabilization method. Based on the results of the present study, localization of JEV C to the cytoplasm alone appears to be the result of fixation/permeabilization with organic solvents (i.e., methanol, ethanol, and acetone), which dehydrate cells and cause protein denaturation and precipitation, thereby masking the epitopes of the nuclear C protein or making them inaccessible to the antibodies. In contrast, the use of cross-linking agents (i.e., paraformaldehyde and formaldehyde) leads to protein cross-linking, which minimizes nonspecific leaching of the nuclear C protein.

Previous evaluation of various fixation/permeabilization methods for flow cytometric analysis of DNA content and nuclear IFA has indicated that in mammalian cells, the molecular architecture of the nucleus is best maintained by fixation with paraformaldehyde followed by Triton X-100 permeabilization [129]; the authors of this evaluation postulated that the paraformaldehyde/Triton X-100 treatment permeabilizes cells but retains their native supramolecular structure, whereas methanol-based fixatives disrupt this structure and randomize the availability of epitopes to antibodies [129]. Therefore, these two fixation/permeabilization procedures can be used as complementary methods for studying the intracellular localization of a protein of interest. Moreover, when a high percentage of acrylamide was used for SDS-PAGE, we were able to detect a low amount of an additional α-C-reactive protein, 1–2 kDa smaller than the full-length C protein, from lysates of JEV-infected BHK-21 cells (data not shown). This observation agrees with a recent report indicating that the host protease cathepsin L is capable of cleaving JEV C between amino acid residues Lys-18 and Arg-19, and this cleavage plays an important role in viral replication in mouse macrophages (RAW264.7) and neuroblastoma (N18) cells, as well as in viral virulence in mice [130].

A set of 10 rabbit antisera against the JEV nonstructural proteins worked well in immunoblotting, detecting the corresponding single or multiple viral proteins in JEV-infected BHK-21 cells, generally in accordance with the flavivirus polyprotein processing model built upon earlier studies with several different flaviviruses [62,106–108,112,131–135]. In the case of NS1/NS1', α-NS1^N-term^ recognized both ~45-kDa NS1 and its frameshifted ~58-kDa NS1', while α-NS1'^FS^ detected only the NS1' protein. These findings are in line with previous results, which have indicated that NS1' is produced by all members of the JE serogroup as a result of a -1 ribosomal frameshift at codons 8–9 of NS2A, creating a 52-amino acid C-terminal extension that contains the N-terminal nine amino acids of NS2A and 43 unique post-frameshift amino acids [79,80,105,136,137]. In our study, the inclusion of the first nine amino acids of NS2A in NS1' was supported by the reactivity of NS1' to α-NS2A.

Like all flaviviruses, JEV encodes three cytoplasmic (NS1, NS3, and NS5) and four membrane-spanning (NS2A, NS2B, NS4A, and NS4B) nonstructural proteins. Unlike the cytoplasmic proteins, all four membrane-spanning proteins, despite their relatively small size, are difficult to express in soluble form as GST-tagged full-length or N/C-terminal half-truncated proteins because of their hydrophobic nature. In each case, we therefore defined a relatively small non-hydrophobic region (55–60 amino acids) to increase its expression and solubility with an N-terminal GST tag. In this approach, we successfully produced three soluble GST-tagged recombinant proteins for NS2A, NS4A, and NS4B (but not for NS2B) and used them to raise the respective antigen-specific rabbit antisera. For NS2B, however, we made multiple attempts to produce a soluble GST fusion protein, with no success, but we were able to generate an NS2B-specific rabbit antiserum by immunizing with a KLH-conjugated synthetic oligopeptide (12 amino acids).

Immunoblotting with these four antisera revealed both expected and unexpected findings: (***i***) The α-NS2A antibody reacted with the ~58-kDa NS1' protein that contains a 9-amino-acid-long that is part of the antigen used to make the antiserum; however, it failed to detect the predicted ~25-kDa NS2A [138–140] that encloses its much longer immunogen of 60 amino acids. Similarly, a previous study showed that in WNV-infected cells, an α-NS2A rabbit antiserum raised against the baculovirus-expressed GST-NS2A reacted in immunoblotting with NS1', but not with the expected NS2A [138]. We speculate that some peculiar and unknown property of JEV/WNV NS2A prevents the protein from being recognized by α-NS2A antibodies. In YFV-infected cells, the viral serine protease can cleave at an internal site in NS2A, yielding a C-terminally truncated form, NS2Aα [108,141]. Mutations at the YFV NS2Aα cleavage site cause a defect in virus assembly [74]. (***ii***) The α-NS2B^Peptide^ antibody reacted only very weakly with the expected ~14-kDa NS2B [139,142] but strongly with an unexpected ~12-kDa protein, suggesting that NS2B is post-translationally modified to yield a form that migrates faster on SDS-PAGE; however, the identity of this modification remains to be determined. (***iii***) The α-NS4A antibody detected a single ~14-kDa protein, and the α-NS4B antibody recognized a ~25/27-kDa doublet, as has been observed for other flaviviruses [106–108,138,139,143]. To our knowledge, the nature of the NS4B doublet has not yet been well defined and is a subject of future studies [107,108].

One of the most surprising findings of our study is that a complementary pair, α-NS3^N-term^ and α-NS3^C-term^, recognized not only the full-length ~69-kDa NS3 but also at least 11 other α-NS3-reactive proteins of ~16–52 kDa, including the two most immunoreactive proteins NS3^p34^ and NS3^p35^. Based on their reactivity with either one or both of these antisera, we conclude that the smaller NS3-related proteins are most likely the N/C-terminally truncated forms of NS3. Although the functional roles of these smaller forms of NS3 are unclear, it is conceivable that they have a distinct function(s) during JEV replication. In previous studies with DENV and TBEV, a C-terminally truncated NS3 product of ~50 kDa was detected in virus-infected cells, as a result of an alternative cleavage within the HEL domain [144–146]. Given its observed molecular weight, the truncated form of DENV/TBEV NS3 corresponds to either the NS3^P50^ or NS3^P52^ protein of JEV NS3. Similarly, we also found in the present study that another complementary pair, α-NS5^N-term^ and α-NS5^C-term^, probed both the full-length ~103-kDa NS5 and six smaller α-NS5-reactive proteins, including NS5^p15^, NS5^p27^, and NS5^p90^. Like JEV NS3, we speculate that these smaller NS5-related proteins are the N/C-terminally truncated forms of NS5. Although it seems less likely, we cannot exclude the possibility that some of the smaller α-NS3- or α-NS5-reactive proteins might be host cellular proteins that were induced by JEV infection and coincidentally cross-reacted with the respective antiserum. With respect to the subcellular localization of JEV NS5, both α-NS5^N-term^ and α-NS5^C-term^ gave a high level of nonspecific background staining when used for IFA of BHK-21 cells, the cell line that we used for JEV infection. Unfortunately, no specific signal above this high background staining was observed in JEV-infected BHK-21 cells (data not shown). However, previous studies have reported that a fraction of NS5 is found inside the nucleus of cells infected with YFV [147], DENV [148], and JEV [149]. In DENV, NS5 has been shown to contain nuclear localization signals recognized by importin β1 and importin α/β [150,151]. The biological relevance of flavivirus NS5 in the nucleus needs to be further studied.

In summary, a major bottleneck in the study of JEV biology has been the lack of a full catalog of antibodies that can specifically recognize all of the viral gene products, which is necessary for understanding the molecular and cell biology of the viral proteins and purifying them for biochemical and immunologic studies. To our knowledge, ours is the first report of the production of a large, nearly complete set of 15 JEV-specific polyclonal rabbit antisera that allows us to profile all of the viral gene products and their related species except NS2A. Our data offer strong support for the accuracy of the current model of flavivirus gene expression. In addition, these antisera are valuable reagents that will open new avenues for the molecular and genetic study of JEV replication and pathogenesis.
